# Therapeutic Efficacy of Transcutaneous Electrical Nerve Stimulation Acupoints on Motor and Neural Recovery of the Affected Upper Extremity in Chronic Stroke: A Sham-Controlled Randomized Clinical Trial

**DOI:** 10.3390/healthcare9050614

**Published:** 2021-05-20

**Authors:** Reem M. Alwhaibi, Noha F. Mahmoud, Hoda M. Zakaria, Walaa M. Ragab, Nisreen N. Al Awaji, Mahmoud Y. Elzanaty, Hager R. Elserougy

**Affiliations:** 1 Rehabilitation Sciences Department, Health and Rehabilitation Sciences College, Princess Nourah Bint Abdulrahman University, Riyadh 11671, Saudi Arabia; rmalwhaibi@pnu.edu.sa (R.M.A.); NFMahmoud@pnu.edu.sa (N.F.M.); 2 Department of Neuromuscular Disorders and Its Surgery, Faculty of Physical Therapy, Cairo University, Cairo 12613, Egypt; dr_hodazakaria@yahoo.com (H.M.Z.); walaa.ragab@cu.edu.eg (W.M.R.); mahmoud.yassen@deraya.edu.eg (M.Y.E.); 3 Department of Physical Therapy, Faculty of Medical Rehabilitation Sciences, Taibah University, Medina 42353, Saudi Arabia; 4 Health Communication Sciences Department, Health and Rehabilitation Sciences College, Princess Nourah Bint Abdulrahman University, Riyadh 11671, Saudi Arabia; NNAlAwaji@pnu.edu.sa; 5 Department of Neuromuscular Disorders and Its Surgery, Faculty of Physical Therapy, Deraya University, New Menya 11159, Egypt; 6 Department of Neuromuscular Disorders and Its Surgery, Faculty of Physical Therapy, Misr University for Science and Technology, Giza 77, Egypt

**Keywords:** stroke, upper extremity, motor impairment, task-specific training, transcutaneous electrical nerve stimulation acupoints, quantitative electroencephalogram

## Abstract

Inability to use the affected upper extremity (UE) in daily activities is a common complaint in stroke patients. The somatosensory system (central and peripheral) is essential for brain reorganization and plasticity. Neuromuscular electrical stimulation is considered an effective modality for improving UE function in stroke patients. The aim of the current study was to determine the therapeutic effects of transcutaneous electrical nerve stimulation (TENS) acupoints on cortical activity and the motor function of the affected UE in chronic stroke patients. Forty male and female patients diagnosed with stroke agreed to join the study. They were randomly assigned to group 1 (G1) and group 2 (G2). G1 received task-specific training (TST) and sham electrical stimulation while G2 received TST in addition to TENS acupoints. Session duration was 80 min. Both groups received 18 sessions for 6 successive weeks, 3 sessions per week. Evaluation was carried out before and after completion of the treatment program. Outcome measures used were the Fugl-Meyer Assessment of the upper extremity (FMA-UE) and the box and block test (BBT) as measures of the motor function of the affected UE. Brain activity of the motor area (C3) in the ipsilesional hemisphere was measured using a quantitative electroencephalogram (QEEG). The measured parameter was peak frequency. It was noted that the motor function of the affected UE improved significantly post-treatment in both groups, while no significant change was reported in the FMA-UE and BBT scores post-treatment in either G1 or G2. On the other hand, the activity of the motor area C3 improved significantly in G2 only, post-treatment, while G1 showed no significant improvement. There was also significant improvement in the activity of the motor area (C3) in G2 compared to G1 post-treatment. The results of the current study indicate that TST only or combined with TENS acupoints can be considered an effective method for improving motor function of the affected UE in chronic stroke patients, both being equally effective. However, TST combined with TENS acupoints proved better in improving brain plasticity in chronic stroke patients.

## 1. Introduction:

Millions of people worldwide are functionally affected by stroke [1,2] with almost 75% of cases occurring in low- to middle-income countries, leading to residual motor disabilities and the need for intensive rehabilitation and eventually, economic, and social burdens on the patient, caregiver and/or family and society [3,4].

Signs and symptoms caused by stroke are variable and depend on the location and severity of injury. Weakness of the upper extremity (UE) is considered one of the most common impairments in stroke patients [5] and occurs in approximately 40% of chronic stroke patients [6]. Despite being a common impairment, the restoration of UE function is usually poor and uncommon the despite presence of various physical therapy modalities that are evidence-based [7].

A main principle of recovery after stroke is promoting brain reorganization, or in other words, neural plasticity, which is a use-dependent process and is constantly modified by somatosensory input, whether exteroceptive or proprioceptive. Repeated sensorimotor stimulation results in a long-lasting increase in the cortical representation of various somatosensory and motor areas in both ipsilesional and contralesional hemispheres [8], which in turn reflects on the functional activity of extremities in stroke patients.

A common physical therapy modality used in rehabilitation of stroke patients is neuromuscular electrical stimulation, which is reported to be able to help control abnormal muscle tone, improve muscle strength, along with joint mobility, and stop or correct contractures that occur because of abnormal joint positions [9]. Examples include transcutaneous electrical nerve stimulation (TENS) applied to peripheral nerve area or acupuncture points and functional electrical stimulation (FES) on the motor points [10]. Acupuncture is one of the main modalities in traditional Chinese medicine (TCM), is used in the treatment of various disorders and has been practiced for over 2000 years. It was a common modality used in the treatment of hemiplegic patients long before the Tang dynasty [11,12] TCM is based on the yin-yang theory, which states that energy called chi flows through organs and helps in maintaining proper health. Acupuncture can help balance energy circulation in different body organs. Electroacupuncture (EA), or electrical acupoint stimulation, is used as a modality of treatment, wherein minimal electric current, like the human bioelectric current, is applied through a needle placed on certain points, called meridian points, of the human body, until a needling sensation is felt by the patient [13]. Some studies reported that the somatosensory stimulation of specific acupoints for 10 min activated cortical and subcortical areas in the cerebral hemisphere and increased motor area representation [14].

Yan et al. (2005) stated that motor recovery after stroke can be promoted by electrical stimulation using surface electrodes applied to peripheral nerves, motor points and acupoints/electroacupuncture [15]. Additionally, studies showed that acupuncture and electroacupuncture have effects like physical exercise on the release of chemical substances in central and peripheral nervous systems (e.g., circulatory and biochemical effects), in addition to an electrophysiological effect, by stimulating afferents in the skin and muscles. Transcutaneous electrical nerve stimulation (TENS) is an example of neuromuscular electrical stimulation used in somatosensory stimulation [16,17,18].

Task-specific training (TST) is a treatment strategy that adopts concepts of motor relearning approach [19] and involves practicing goal-directed, functional everyday tasks such as holding a cup in an environment like the original one [20,21]. TST promotes the recovery of paretic muscles in stroke patients [22,23,24] and can be considered more effective than traditional physical therapy approaches [25].

Quantitative electroencephalography (QEEG) is a noninvasive, affordable neurophysiological technique that measures brain activity during various stroke recovery stages [26]. It is divided into four standard frequency bands, which are delta (1–4 Hz), theta (4–8 Hz), alpha (8–12 Hz) and beta (12–30 Hz). QEEG can provide valuable information regarding the effectiveness of various physical therapy modalities in the management of stroke patients [3,27].

As mentioned previously, some clinical studies reported that repeated somatosensory stimulation could enhance neural plasticity, but to our knowledge, in no studies were both clinical evaluation and EEG brain mapping used to investigate the effect of sensory stimulation (i.e., TENS) combined with TST on motor impairment in stroke patients.

Therefore, the present study aimed to determine the therapeutic effects of TENS acupoint on cortical activity and motor function of the affected UE in chronic stroke patients. It was hypothesized that TENS acupoints will not have any therapeutic effects on the functional performance of the affected UE in chronic stroke patients nor on the motor cortical activity of the ipsilesional hemisphere.

## 2. Subjects and Methods

### 2.1. Study Design

This is a sham-randomized clinical study. The ethical committee of the Faculty of Physical Therapy at Cairo University, Egypt, reviewed and approved this clinical study (P.T.REC/012/002153). Fifty-one male and female patients diagnosed with stroke (*n* = 51) were recruited from the Outpatient Physical Therapy Clinic—Faculty of Physical Therapy at Cairo University. After a throughout evaluation, (*n* = 11) were omitted for not agreeing with the conditions of the study along with other reasons, and (*n* = 40) were included in the study. The remaining 40 patients were randomly assigned to groups (G1 and G2).

In this study, 19 men and 21 women aged 40–65 years, diagnosed with first-time ischemic/hemorrhagic stroke by their treating neurologists and confirmed by MRI scans, participated in the study. All patients were reported medically stable by their neurologist with a stroke incidence range between 6 and 24 months, scored (>25) on the mini-mental state examination (MMSE); the affected UE scored [1] on the modified Ashworth scale (MAS), and patients had normal sensation of the affected UE, as measured by the Nottingham Sensory Assessment (NSA) scale (score 2 for tactile sensation and kinesthesia and score 3 for stereognosis).

The exclusion criteria were intolerance to TENS acupoints; UE sensory deficits attributable to nonstroke pathology, such as diabetes or peripheral neuropathy; prestroke orthopedic or neurological injury that caused the somatosensory or motor impairment of the affected UE; presence of receptive (sensory) or expressive (motor) aphasia; impaired vision; presence of visuospatial or unilateral spatial neglects; uncontrolled poststroke seizures; or participation in another experimental rehabilitation project during the duration of treatment. The treatment protocol was explained and demonstrated to each patient, after which each patient was asked to sign a consent form after reading it carefully. Each patient was advised they can withdraw from the study any time they need to.

### 2.2. Randomization

A random number software (http://www.randomization.com) (accessed on 1 January 2021) was used to equally randomize patients to two equal groups (G1 and G2). G1 received TST and sham electrical stimulation, while G2 received TST and TENS acupoints.

### 2.3. Sample Size

The sample size for the current study was calculated using G*POWER statistical software (version 3.1.9.2; Franz Faul, Universitat Kiel, Germany), which indicated a required sample of 40 patients for the whole study (α was set at 0.05, β at 0.2, size effect 0.91 and allocation ratio N2/N1 = 1). 

### 2.4. Clinical Examination

The Fugl-Meyer Assessment of the upper extremity (FMA-UE), box and block test (BBT), and quantitative electroencephalogram (QEEG) were applied twice in week 0 (pre-treatment) and week 7 (post-treatment) by a well-trained and experienced physical therapist for the FMA-UE and BBT and by a specialized neurophysiologist for QEEG.

#### 2.4.1. Fugl-Meyer Assessment of the Upper Extremity (FMA-UE)

The FMA-UE is a measure of stroke impairment. It includes 33 items that measure reflexes, movement patterns (synergies), coordination, sensation, and the joint range of motion (ROM) of the affected UE. Each item is scaled from 0–2 (2 for full task performance, 1 for partial task performance and 0 no performance of the task) [28,29,30,31]. The maximum achievable score is 66, indicating full recovery, and the assessment takes up to 30 min to finish [29,32]. The FMA-UE has a high inter-rater and test–retest reliability [17,32]. In the current study, the FMA-UE was applied to the affected extremity, and the score achieved by each patient was reordered.

#### 2.4.2. Box and Block Test (BBT)

The BBT is a test of unilateral gross manual dexterity wherein a pick-up and release action is performed by the fingers [33]. BBT has a test–retest reliability > 0.9 [34,35].

In the current study, each patient was seated in a quiet room in front of a table with adjustable height. A wooden box containing 150 wooden cubes of similar shapes was placed on the table. Patients were instructed to pick up one cube at a time with the tip of the index and middle finger of the affected UE, move it over the separating partition of the box and drop it into the opposite compartment for 1 min [36]. Before the test began, each patient could rehearse the pick-up and release movement of the cubes for approximately 15 s. A stopwatch was used for monitoring time. The higher the score, the better is the gross manual dexterity.

#### 2.4.3. Quantitative Electroencephalogram (QEEG)

A digital EEG-EP Multifunction System 10–20 Channel (EBNeuro, Florence, Italy/Mizar-Pc Peripheral System CE Version-B9800037800) was used to record EEG signals from the ipsilesional hemisphere (right or left). Patients were asked to assume supine lying position with eyes closed (to minimize artifacts from eye movements or any visual feedback) and to stay awake during the exam. A cap with 64 Ag/AgCl scalp monopolar electrodes was placed, based on the international 10/20 system used to record EEG signals. Impedances were kept below 5 kΩ [37].

Prior to EEG testing, each patient was given instructions regarding the evaluation process by the same neurophysiologist performing the test. The recorded data then was transformed into the mapping program to perform a spectral analysis. The data recorded and analyzed was the peak frequency of alpha waves. The brain function and plasticity of motor area C3 in the ipsilesional hemisphere were measured by QEEG for both groups.

### 2.5. Therapeutic Interventions and Procedures

Patients in both groups received a total of 18 sessions, over a period of 6 successive weeks, 3 sessions per week, by an experienced and well-trained physical therapist. The session duration was 80 min. Patients in G1 received TST for 60 min and sham electrical stimulation for 20 min, while patients in G2 received TST for 60 min and TENS acupoints for 20 min. Patients in both groups practiced the same exercises at home.

#### 2.5.1. Task-Specific Training (TST)

Six TST exercises, based on the motor relearning approach [19], were practiced as follows:

1. mimicking drinking water from a glass;
2. holding a cup of water and raising it to a level of 90° shoulder flexion while maintaining the elbow joint extended;
3. picking up five tennis balls and moving them from a table to a box;
4. polishing a table with a towel, in various directions, while keeping the elbow joint extended;
5. holding a cone and moving it from a table to a shelf;
6. brushing the hair using a comb.

The duration of a TST session was 60 min. Each session started with warm-up exercises for 10 min followed by 50 min of TST. Each task was repeated approximately 10 to 20 times, for 1 to 5 sets, or alternatively for 2 to 5 min. A 2 min rest period after every 15 min of practice was allowed. Before commencing the exercise session, tasks were demonstrated to each patient using the nonaffected UE. During the session, tasks were performed passively or with therapist help (active assisted), based on each patient’s ability. Speed, distance and/or resistance gradually increased, based on patients’ abilities. The treating therapist provided various types of feedback, such as verbal, visual or proprioceptive, and ensured tasks were performed properly [24,38,39].

#### 2.5.2. Transcutaneous Electrical Nerve Stimulation (TENS) Acupoints

Electrical stimulation was applied to patients in both groups from a supine lying position. Patients in G1 received sham electrical stimulation using self-adhering electrodes for 20 min with minimal intensity that caused no sensory or motor stimulation on the same acupoints points as in G2.

Patients in G2 received 20 min of TENS acupoints (100 Hz, 0.2 ms, square pulses, 2 to 3 times the sensory threshold) from a TENS stimulator (Phyaction 785, serial number: 2753 manufactured by Uniphy B.V., Eindhoven, The Netherlands). Self–adhering electrodes were placed on the affected UE over two points used in traditional Chinese medicine as locations for acupuncture. Those two points relate to the large intestine (LI-11 and LI-4) [40].

In Chinese medicine, acupuncture points LI4 (He Guo) and LI11 (Qu Chi) are considered effective points [41]. With the elbow joint flexed at a right angle, acupoint Quchi (LI11) is localized midway between the lateral end of the transverse cubical crease and the lateral epicondyle of the humerus [42]. It is used in paralysis, pain and motor affection of the shoulder joint and swelling and pain in the knee, among others [43]. The second point, LI4 (He Guo), is located at the base of the first and second metacarpal bones on the dorsum [44]. It is indicated for hemiplegia, finger spasm, and pain in the arm, among others [45].

### 2.6. Statistical Analysis

Statistical Package for Social Science (SPSS) Software (version 19) (IBM, Armonk, NY, USA) for Windows was used for analysis. Differences between variables were considered significant at *p* ≤ 0.05. Cohen’s *d* and effect size (*r*) were calculated for each parameter in this study. Mean and standard deviation were calculated for all variables of the study. An analysis of demographic data and baseline clinical characteristics was carried out using an unpaired *t*-test and the chi-square test. The difference between and within G1 and G2 in QEEG was calculated using a paired *t*-test, while Wilcoxon and Mann–Whitney tests were used for comparison between the scores of the FMA-UE and BBT within and between both groups.

## 3. Results

**Figure 1:**
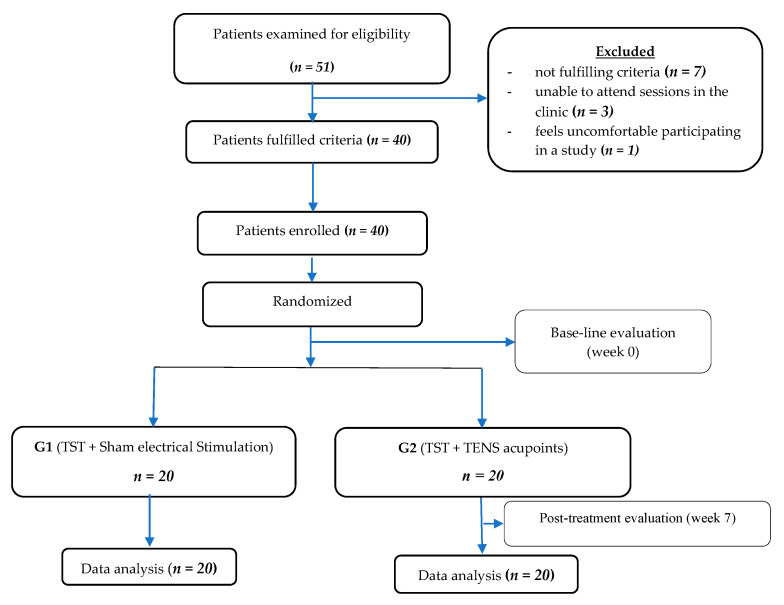
Diagram showing the flow of the study.

Fifty-one stroke patients were screened for eligibility. 11 did not match the specified inclusion criteria of the study, and the remaining 40 patients in both groups completed the study (Figure 1).

### 3.1. Demographic Data and Baseline Clinical Characteristics

No statistically significant pretreatment difference (*p* > 0.05) was present between G1 and G2 in age, gender, height, weight, type (ischemic or hemorrhagic) and duration (months) of stroke. Cohen’s *d* had a small effect size for all parameters (*r* < 1) (Table 1).

### 3.2. Clinical Scales

No significant difference was present between G1 and G2 in pretreatment scores of the FMA-UE and BBT, while, post-treatment, there were significant improvement within both G1 and G2, but no significance difference between the groups was present (*p* < 0.05) Cohen’s *d* had a small effect size for all parameters (*r* < 1) (Table 2 and Table 3).

### 3.3. Quantitative Electroencephalogram (QEEG)

There was no significant difference between G1 and G2, pretreatment, in the peak frequency of alpha waves of motor area (C3). On the other hand, there were significant improvement in G2, post-treatment (*p* = 0.0005), and in G2 compared to G1 (*p* = 0.001) post-treatment. Cohen’s *d* had a small effect size for all parameters (*r* < 1) (Table 4).

## 4. Discussion

This study showed that 6 weeks of TST only or combined with TENS acupoints improved motor function of the affected UE in chronic stroke patients, which was reflected in the scores on the FMA-UE and BBT in G1 and G2. However, when comparing both groups together, neither TST alone nor TST in addition to TENS acupoints proved better in improving FMA-UE and BBT scores of the affected UE post-treatment, or, in other words, the functional performance of the affected UE was not improved. Effects were minimal and a small effect size was exhibited.

TSTs improve functional abilities using skilled repetitions of a specific task, rather than the standard stretching, or strengthening exercises usually practiced in rehabilitation programs [24,46]. Practicing tasks like basic ADLs [47] can result in better neural plasticity. An effective TST treatment program must be challenging both physically and intellectually, gradually progress from easy to hard tasks and finally, challenge the patient’s interests. TSTs stimulate ipsilesional sensory and motor brain areas that are anatomically and physiologically connected to the contralesional hemisphere and/or recruit supplementary brain areas of the brain, which is one of the main concepts of neural plasticity used in rehabilitation of stroke [24,39].

Additionally, applying TENS through certain acupuncture points (electroacupuncture) activates small diameter fibers (Aα) arising from muscles (ergo receptors), which results in phasic muscle twitches [9]. Ergo receptors are mechanical receptors present in skeletal muscles, tendons, and joint structures. Their main function, along with other receptors, is the facilitation of joint proprioception and kinesthetic senses [48]. Proprioception is the awareness of the relative position of various body parts in relation to space, derived from muscle spindles, GTO, joint and cutaneous receptors. Improving proprioception results in improvement in movement control and function [49]. Several studies on electroacupuncture treatment investigated the effects of this type of treatment on spasticity or increased muscle tone, which was not the case in the current study, since the sample selected had minimal increase of muscle tone measured by MAS [13,50,51,52,53,54,55]). Still, we can postulate, that even though spasticity was mild in the sample selected in the current study, the slight improvement in muscle tone might reflect better motor performance of the affected UE, especially in fine movements.

On the other hand, a study by Au-Yeung et al. (2014) [8] reported that electrical acupoint stimulation during acute stroke appeared to “produce greater and longer lasting hand grip and pinch strength improvements than administering conventional rehabilitation alone”.

The current study showed positive significant improvement in the activity of motor area (C3) in G2, post-treatment, which may be attributed to the fact that TST is a rehabilitation strategy that involves practicing various functional movements that are goal-directed in an environment like real life. This helps patients in developing the best control strategies for managing various movement disorders, not just stroke [56]. TSTs can cause improvements in the structural integrity of the brain that leads to cognitive improvement and is highly effective in the process of brain neurogenesis compared to simple or passive exercise alone. Another very important aspect of TST is it involves the use of external feedback, such as visual input, which helps in learning and the memorization of movement patterns [57]. Richards et al. (2008) [58] reported that the reshaping of the primary motor cortex in both ipsilesional and contralesional brain areas is dependent on experience, which occurs in TST after the repetition of various tasks.

The improvement seen in G2 compared to G1 in terms of brain activity might also be related to the fact that sensory and motor inputs result in constant changes in cortical representation (i.e., physiological reorganization) that occurs postinjury in various areas of the brain. Evidence showed that afferent sensory input by electrical devices such as TENS stimulate both sensory and motor brain areas, which has an excellent effect on functional improvement poststroke [59,60]. Another interesting explanation for the increased excitability of motor area (C3) in G2 compared to G1 is cutaneous electrical stimulation increasing blood circulation in the sensorimotor cortex and stimulating large diameter A afferent motor and sensory fibers [61] However, the heterogeneity of patients, regarding the type of stroke (ischemic versus hemorrhagic), and the difficulty in fixing one or two tasks for all patients in the current study (i.e., each patient practiced the task based on their ability) made it difficult to reach conclusive facts about the effectiveness of TST and TENS acupoints on the functional recovery of the affected UE in chronic stroke patients.

Still, the findings of the present study might indicate that somatosensory stimulation in the form of TENS acupoints and TST facilitates neural plasticity, which reflects the motor function of the affected UE in chronic stroke patients. Thus, physical therapists can use this therapeutic program as one of the many interventions used in the rehabilitation of stroke patients.

### Limitations

The limitation of the present study is that improvement of the affected UE function cannot be solely based on improved patterns of cortical activation and should also be reflected clinically (as measured by functional scales). Therefore, further studies using other techniques of noninvasive brain imaging technology, such as functional magnetic resonance imaging (fMRI), should be conducted to observe cortical reorganization following TST and TENS in stroke patients and how it corresponds to improvements in the affected UE. Probably, there is also a need for more homogeneous patient groups, in terms of cause of stroke (ischemic or hemorrhagic) and lesion site. Finally, the long-term effect of electroacupuncture/electrical acupoint needs to be investigated. Additionally, the current study did not consider the hand dominance of patients or the side of affection.

## 5. Conclusions

Based on the findings of the present study, it is possible to reach the conclusion that using TST only or added to TENS acupoints improves the motor function of the affected UE in chronic stroke patients. Additionally, the present study provides evidence that adding TENS acupoints to the treatment program improves the brain activity of motor areas. Yet, further investigations are required to confirm these findings.

## Figures and Tables

**Table 1:** Demographic and baseline clinical characteristics in both groups, pretreatment.

Demographic Data	G1 (*n* = 20)	G2 (*n* = 20)	*p* Value	Cohen’s *d*	Effect Size (*r*)
Age (years)	51.8 ± 5.7	52.5 ± 4.9	0.584 ^a^	−0.132	−0.066
Gender (male/female) Type (infarction/hemorrhage)	11/9 12/8	8/12 13/7	0.457 ^b^ 0.652 ^b^	- -	- -
Height (cm)	166.1 ± 7.21	164.7 ± 8.34	0.784 ^a^	0.180	0.089
Weight (kg)	65.2 ± 9.42	67.3 ± 11.31	0.651 ^a^	−0.201	−0.100
Duration of stroke (month)	21.22 ± 2.09	22.21 ± 3.07	0.949 ^a^	−0.377	−0.185

^a^ Unpaired *t*-test, ^b^ Chi-squared test.

**Table 2:** Fugl-Meyer Assessment of the upper extremity (FMA-UE) of the affected UE in both groups.

FMA-UE	G1	G2	*p* Value	Cohen’s *d*	Effect Size (*r*)
Pre-test	24.8 ± 3.05	24.8 ± 2.65	0.999 ^b^	0	0
Post-test	27.2 ± 3.14	28 ± 1.99	0.4116b ^b^	−0.304	−0.150
*p* Value	0.0001 ^a,^*	0.0001 ^a,^*	-	-	-

^a^ Wilcoxon test; ^b^ Mann–Whitney test; * Significant at *p* < 0.05.

**Table 3:** Box and block test (BBT) of affected UE in both groups.

BBT	G1	G2	*p* Value	Cohen’s *d*	Effect Size (*r*)
Pre-test	23 ± 21.9	14 ± 15.5	0.517 ^b^	0.474	0.231
Post-test	38 ± 22.1	20 ± 12.3	0.790 ^b^	1.006	0.449
*p* Value	0.005 ^a,^*	0.002 ^a,^*	-	-	-

^a^ Wilcoxon test; ^b^ Mann–Whitney test; * Significant at *p* < 0.05.

**Table 4:** Peak frequency of alpha wave of motor area (C3) of ipsilesional hemisphere in both groups.

QEEG (C3)	G1	G2	*p* Value	Cohen’s *d*	Effect Size (*r*)
Pre-test	8.326 ± 0.13	8.501 ± 0.1	0.394 ^b^	1.363	0.563
Post-test	8.298 ± 0.03	9.443 ± 0.4	0.001 *^,b^	−4.037	−0.896
*p* Value	0.886 ^a^	0.0005 *^,a^	-	-	-

^a^ Paired *t*-test; ^b^ Unpaired *t*-test; * Significant at *p* < 0.05.

## Data Availability

The data presented in this study are available on request from the corresponding author.
